# First-Trimester Clinical Characteristics and Pregnancy Outcomes in Women with Recurrent Pregnancy Loss

**DOI:** 10.3390/jcm14165797

**Published:** 2025-08-16

**Authors:** Cristina Trilla, Judit Platero, Núria Camprubí, Josefina Mora, Cristina Luna, Daniel Oros, Elisa Llurba

**Affiliations:** 1 Department of Obstetrics and Gynecology, Institut d’Investigació Biomèdica Sant Pau-IIB Sant Pau, Hospital de la Santa Creu i Sant Pau, 08025 Barcelona, Spain; ctrilla@santpau.cat (C.T.); jplaterod@santpau.cat (J.P.); ncamprubi@santpau.cat (N.C.); 2 Faculty of Medicine, Universitat Autònoma de Barcelona, 08193 Barcelona, Spain; jmora@santpau.cat; 3 Spanish Network in Maternal, Neonatal, Child and Developmental Health Research (RICORS-SAMID, RD24/0013/0001), Instituto de Salud Carlos III, 28040 Madrid, Spain; 4 Women and Perinatal Health Research Group, Institut de Recerca (IR SANT PAU), Sant Quintí 77-79, 08041 Barcelona, Spain; 5 Department of Biochemistry, Hospital de la Santa Creu i Sant Pau, 08025 Barcelona, Spain; 6 Obstetrics Department, Aragon Institute of Health Research (IIS Aragon), Hospital Clínico Universitario Lozano Blesa, 50009 Zaragoza, Spain; cristina.luna93@gmail.com (C.L.); danoros@gmail.com (D.O.)

**Keywords:** recurrent pregnancy loss, first trimester, maternal characteristics, biochemical markers, uterine artery pulsatility index, preeclampsia, screening

## Abstract

**Objective**: To describe first-trimester maternal, biochemical, biophysical, and ultrasound characteristics in women with recurrent pregnancy loss (RPL) compared to women without RPL. **Methods**: This was a retrospective cohort study analyzing data from 4440 pregnant women, including 142 women with previous RPL. Maternal and pregnancy characteristics, first-trimester biochemical markers, biophysical assessments, early-onset preeclampsia (EOPE) risk, and perinatal outcomes were compared. **Results**: Women with RPL were older (37.8 vs. 34.0 years, *p* < 0.001) and had higher rates of antiphospholipid syndrome (4.9% vs. 0.9%, *p* < 0.001), other thrombophilias (5.6% vs. 0.8%, *p* < 0.001), and thyroid disorders (14% vs. 7.5%, *p* = 0.010) than women without RPL. First-trimester uterine artery pulsatility index (UtA-PI) values, pregnancy-associated plasma protein-A (PAPP-A) levels, mean arterial pressure, and final risk for EOPE were comparable between groups. However, the RPL group had higher rates of very high risk for PE (10.6 vs. 5.1, *p* = 0.011). Likewise, second-trimester UtA-PI was higher in this group (1.10 vs. 1.01, *p* = 0.045). Aspirin and low molecular weight heparin prophylaxis were more frequent in women with RPL (23.8% vs. 9.6%, *p* < 0.001; 14.7% vs. 0.1%, *p* < 0.001). Regarding perinatal outcomes, we found a higher incidence of second-trimester intrauterine demise in the RPL group (6.4% vs. 1.4%, *p* = 0.011), with no other differences observed in the remaining outcomes. **Conclusions:** Women with RPL exhibit distinct maternal characteristics and worse pregnancy outcomes, although first-trimester markers do not seem to significantly differ from findings in women without RPL. These findings underscore the importance of tailored screening and intervention protocols to improve perinatal outcomes in this high-risk population.

## 1. Introduction

Spontaneous miscarriage, which occurs in about 15–20% of clinically recognized pregnancies, represents the most prevalent complication during early pregnancy [1]. Recurrent pregnancy loss (RPL) has been historically defined as the loss of three or more consecutive pregnancies before the fetus reaches viability. However, recent guidelines define RPL as two or more pregnancy losses, consecutive or not [2]. We adopted this updated definition to reflect current clinical practice. RPL affects approximately 3–5% of couples attempting to conceive, and its incidence is particularly high in the first trimester of pregnancy [3,4], posing significant emotional and physical challenges to affected women. While associated etiologies are multifactorial, including gynecological, anatomical, genetic, hormonal, immunological, and environmental factors, many cases remain unexplained despite comprehensive evaluations [5]. This highlights the complexity of miscarriage diagnosis and the pregnancy management of women with previous RPL.

Maternal characteristics of women with RPL have been extensively described. Previous studies have identified advanced maternal age, thrombophilia, and autoimmune disorders as significant risk factors for RPL [6]. Women with RPL are also at an increased risk of adverse obstetric outcomes, such as preeclampsia (PE), preterm birth (PTB), and intrauterine growth restriction (IUGR). Recent research has shown that women with a history of RPL are 2–3 times more likely to develop gestational hypertension or preeclampsia, and they exhibit a 60% increased risk of PTB, compared to the general obstetric population [7,8,9]. The pooled odds ratios of these complications seem to progressively increase with increasing number of miscarriages, indicating a dose-response pattern and supporting the presence of a biological gradient [8]. Furthermore, the odds for IUGR are two times higher in these women [10], underscoring the potential long-term impact of RPL on maternal and fetal health.

Despite maternal characteristics, obstetric outcomes, and long-term health outcomes of women with RPL having been previously described, limited data exist on first-trimester characteristics between women with and without RPL. Miscarriage and placental dysfunctional disorders share, to some extent, the same pathophysiology. Thus, a history of RPL might be associated with an increased risk of PE and placental insufficiency disorders. As the pyramid of care in obstetrics has shifted toward improved stratification of risk in the first trimester, understanding these differences is essential for optimizing pregnancy management and maternal and fetal outcomes in women with previous RPL. This study aims to describe the first-trimester maternal, biochemical, biophysical, and ultrasound characteristics of women with RPL compared to women without a history of RPL.

## 2. Materials and Methods

This was a prospective observational cohort study that included women with a singleton pregnancy who underwent ultrasound follow-ups in the Prenatal Diagnosis Unit of Hospital de la Santa Creu i Sant Pau between January 2020 and December 2022. Women with a singleton pregnancy and a fetus with a crown-rump length (CRL) ranging from 45 to 84 mm were invited to participate. Women were withdrawn from the study in case of a fetal chromosomal or genetic abnormality, or in case of a major fetal anomaly diagnosed during pregnancy or at birth. This study is an unplanned subanalysis of a larger research study aimed at determining the effectiveness of a sequential screening strategy for PE in the first trimester. This study was approved by the Ethics Committee of the Institutional Review Board at Hospital de la Santa Creu i Sant Pau (protocol code IIBSP PRE 2018 08; date of approval: 28 March 2018) and was registered with ClinicalTrials.gov, number NCT04767438. All participants gave their written consent to participate.

### 2.1. Maternal and Pregnancy Characteristics

Baseline maternal characteristics were documented, including age, ethnicity, body mass index (BMI), smoking status, and medical history (diabetes, hypertension, kidney disease, autoimmune and thyroid disorders, and inherited or acquired thrombophilia, such as antiphospholipid syndrome (APS)). Women with two or more pregnancy losses of a clinically confirmed pregnancy, whether consecutive or not, were classified as women with previous RPL. Additionally, the following pregnancy-related information was recorded: type of conception, parity, use of aspirin or low-molecular-weight heparin (LMWH) during pregnancy, gestational age (GA) at the time of the first-trimester blood analysis, and GA at the time of the first and second-trimester ultrasounds.

### 2.2. First-Trimester Variables

First-trimester variables included biochemical and biophysical variables. Among biochemical variables, pregnancy-associated plasma protein-A (PAPP-A) and placental growth factor (PlGF) were assessed. Serum levels of PlGF and PAPP-A were measured using fully automated electrochemiluminescence immunoassays on a Cobas e 601 analyzer (Roche Diagnostics, Tokyo, Japan). Multiples of the median (MoM) values for first-trimester UtA-PI, PAPP-A, and PlGF were derived from locally established medians, employing a multivariate Gaussian distribution model validated in our population [11].

Biophysical variables included mean arterial pressure (MAP) and uterine artery pulsatility index (UtA-PI). Systolic (SBP) and diastolic blood pressure (DBP) were measured on the day of the first ultrasound using a calibrated Tensoval Duo Control device (Hartmann AG, Heidenheim, Germany). Blood pressure was recorded once after a brief resting period, with the woman seated, as in our standard clinical practice. MAP was calculated using the formula: MAP = (SBP + 2 × DBP)/3. UtA-PI was assessed transabdominally during the first and second trimesters using a standardized methodology [12].

### 2.3. Obstetric Outcomes

First-trimester early-onset PE screening was estimated using a previously described multivariate method including maternal factors, biochemical, and biophysical variables [13]. In the first stage of screening, three risk groups were defined: if risk is >1/50, women were identified as very high risk and aspirin prophylaxis was recommended; if risk is between 1/51 and 1/500 women were defined as intermediate risk and required a second stage of evaluation, and if risk is <1/501 women were identified as low risk. In a second stage, PlGF was determined in the intermediate-risk group, finally stratifying women as low- or high-risk. The final results of this screening were recorded for analysis and made available to all patients. However, only 10 women in the RPL group had an intermediate risk at the first stage of screening, and thus, a comparison of PlGF results between groups was not performed. Perinatal outcomes, including second-trimester pregnancy loss (below 28 weeks), stillbirth (above 28 weeks), PE, birthweight, gestational age at delivery, and mode of delivery, were also documented.

All maternal and pregnancy characteristics, biochemical and biophysical variables, screening results, and perinatal outcomes were extracted from clinical records. Data were analyzed using descriptive statistics. Continuous variables were expressed as means (standard deviations) or medians (interquartile ranges) and compared using t-tests or Mann–Whitney U tests, where appropriate. Categorical variables were expressed as frequencies (%) and analyzed using chi-square or Fisher’s exact tests. A *p*-value < 0.05 was considered statistically significant.

## 3. Results

A total of 4476 women were included in the study. Of those, 36 were withdrawn due to exclusion criteria, leaving a final study population of 4440 women, with 142 of them having a history of RPL (3.2%).

Women with RPL were significantly older (37.8 ± 4.6 vs. 33.9 ± 5.2 years, *p* < 0.001). They had a higher prevalence of antiphospholipid syndrome (4.9% vs. 0.9%, *p* < 0.001), other thrombophilias (5.6% vs. 0.8%, *p* < 0.001), thyroid disorders (14.1% vs. 7.5%, *p* = 0.009), and use of assisted reproductive technologies (16.2% vs. 9.9%, *p* = 0.022), including egg donation. In this study, the rate of nulliparity was higher in women without a history of RPL (43% vs. 56.7%, *p* = 0.001). Among obstetric outcomes in previous pregnancies, the rates of previous PTB, PE, IUGR, and gestational diabetes were higher in the RPL group. However, only the differences for gestational diabetes were statistically significant (1.7% vs. 4.2%, *p* = 0.042). These results are summarized in Table 1.

Table 2 depicts the results regarding the comparison of first-trimester variables and PE risk assessments between groups. There were no significant differences in biochemical markers such as PAPP-A (1.10 vs. 1.04 MoM, *p* = 0.691). No differences were found in the first-trimester biophysical variables included in the study. There were no statistical differences in the final high-risk rate for early-onset PE, although this rate was higher in the RPL group (13.4% vs. 9.5%, *p* = 0.146). However, the rate of women with a very high risk of PE (>1/50) was significantly higher in the RPL group (10.6% vs. 5.1%, *p* = 0.011). Regarding the second-trimester UtA-PI, results showed a significantly higher UtA-PI in women with RPL (1.10 vs. 1.01, *p* = 0.045). Women with RPL were more likely to receive aspirin (23.9% vs. 9.5%, *p* < 0.001) and LMWH (14.8% vs. 0.1%, *p* < 0.001) prophylaxis during pregnancy.

Perinatal results were available for 3902 participants (87.9%). Pregnancy outcomes were similar between groups, with no significant differences in birth weight, overall PE rates (4.8% vs. 4.5%, *p* = 0.824), or cesarean delivery rates (28% vs. 24.8%, *p* = 0.449). However, women with RPL had a significantly higher rate of second-trimester intrauterine fetal demise (6.4% vs. 1.4%, *p* = 0.001). Among those cases, the mean GA at demise was 17.3 (3.1) weeks. There were 11 cases of severe IUGR before 22 weeks. Only 4 of the cases ending in second-trimester pregnancy loss had a first-trimester high-risk result at the PE screening. Prophylactic aspirin had been prescribed in 5 cases, with one patient also taking LMWH. Finally, women with RPL delivered one week earlier than women without a history of RPL, although this difference did not reach statistical significance. The results are presented in Table 3.

## 4. Discussion

This study’s results highlight significant differences in maternal characteristics and pregnancy outcomes between women with and without RPL. Although no significant differences were found in the first-trimester biochemical and biophysical variables analyzed, women with RPL exhibited higher rates of very high risk of early-onset PE and higher rates of second-trimester uterine demise, underscoring the need for tailored obstetric care in this population.

Our study confirmed several associations between maternal factors and RPL. For instance, women with RPL had a more advanced maternal age and a higher prevalence of thrombophilia and thyroid disorders. This is consistent with the previous literature and underscores advanced maternal age as a significant risk factor for both miscarriage and poor obstetric outcomes [14,15]. Age-related decline in oocyte quality and increased aneuploidy rates likely contribute to these outcomes [16]. However, even after excluding pregnancies with confirmed chromosomal or genetic abnormalities, the rate of intrauterine demise remained higher in women with previous RPL. This finding suggests that other factors may play a critical role in the pathophysiology of pregnancy loss in women with RPL beyond the first trimester, highlighting the need for a structured and comprehensive evaluation in this population.

We found a strong association between APS and other thrombophilia with RPL, which also aligns with the previous evidence [17]. APS is one of the most well-documented causes of RPL, with multiple studies confirming its role in pregnancy loss through placental vascular dysfunction [18]. Consistent with these findings, the use of aspirin and LMWH prophylaxis was significantly higher in the RPL group. These therapies have been shown to improve live birth rates in women with APS by preventing antiphospholipid antibodies from binding to the trophoblast, preventing complement activation, promoting trophoblastic invasiveness, and mitigating placental thrombotic events [19]. However, treatment rates with aspirin or LMWH in our study were higher than expected and cannot be attributed to maternal risk factors or preeclampsia screening alone. This corroborates the widespread use of prophylactic treatments in women with RPL in clinical practice. However, while these interventions are widely recommended, their efficacy in improving outcomes in women with unexplained RPL is highly controversial and remains a topic of ongoing research [20,21]. This reflects current clinical practice but may have mitigated the risk assessment of adverse outcomes, such as PE or IUGR, in our study. This constitutes a potential confounding factor that limits the interpretation of outcome differences between groups. Future studies should consider stratified analyses based on treatment exposure or exclude treated cases to better isolate the effect of RPL itself.

Similarly, we found a higher prevalence of thyroid disorders in the RPL cohort. This reinforces the importance of thyroid function screening in this population. Subclinical hypothyroidism and thyroid autoimmunity are recognized contributors to miscarriage, likely due to impaired endometrial receptivity [22,23]. Our findings highlight the necessity of optimizing thyroid function preconceptionally and during early pregnancy to improve outcomes.

Regarding the first-trimester variables included in PE screening, our results showed comparable levels of PAPP-A between groups. This contrasts with some prior studies suggesting these markers are potential predictors of adverse pregnancy outcomes [24,25]. No further differences were observed in UtA-PI, and consequently, the final rates of high risk for PE were comparable between groups. However, RPL is not a maternal factor included in any of the multivariate systems that have been proposed to screen for PE. Our study differs from the previous research in several key aspects. We evaluated first-trimester biochemical and biophysical markers at the time of routine preeclampsia screening. In contrast, many prior studies assessed biomarker levels either outside of pregnancy or at very early gestational stages, frequently in non-viable pregnancies or immediately following miscarriage. Thus, our study focuses on women with a history of RPL who have now achieved an ongoing pregnancy. This allows us to explore whether their first-trimester profile differs from that of women without RPL, offering a novel perspective that has not been the focus of earlier studies.

In our study, women with prior RPL had significantly higher rates of very high risk of PE and higher UtA-PI in the second trimester. Thus, even before second-stage screening using PlGF, women with RPL already exhibited a high-risk profile, supporting the need for early and tailored interventions in this population. This suggests that this population should be considered at higher risk for adverse outcomes regardless of first-trimester results. While the mechanistic link between RPL and later uteroplacental insufficiency is not fully understood, shared pathophysiological pathways such as impaired trophoblastic invasion, endothelial dysfunction, maternal immune response, or subclinical inflammation may play a role. The clinical usefulness of additional biomarkers addressing these biological processes in this population should be further investigated [25].

The association between RPL and subsequent adverse pregnancy outcomes is a subject of ongoing research and debate. Some studies have identified a significant correlation between RPL and increased risks of complications such as pre-eclampsia, preterm birth, and small-for-gestational-age infants [26]. Conversely, other research has suggested that many women with a history of RPL can experience successful pregnancies without significant complications [27,28]. These conflicting findings indicate that, while there is evidence supporting an increased risk of adverse outcomes in women with RPL, the magnitude of this risk may vary depending on individual circumstances and obstetric care. In our study, we found a lower rate of nulliparity in the RPL group. This is notable and may reflect a subgroup of women who experienced successful pregnancies before or after presenting with RPL. This could suggest that this population presents different underlying pathophysiological mechanisms compared to women with primary RPL. Moreover, prior parity may be protective for certain outcomes and could partially explain the lack of significant differences in outcomes such as PE and IUGR, despite increased maternal risk factors.

The higher rate of intrauterine fetal demise in women with RPL found in our study is particularly concerning. This indicates that pregnancy risk in women with RPL extends beyond the first trimester. It underscores the need for advanced monitoring and tailored care throughout pregnancy in this population. Increased antenatal surveillance, including serial assessments of placental function, fetal growth, and well-being, may be beneficial in mitigating these risks. Likewise, understanding the underlying cause of miscarriage may lead to early interventions and improved outcomes.

This study’s strengths include its prospective design and the use of multivariate screening for identifying high-risk pregnancies, ensuring robust and clinically relevant findings. Another significant strength is the comprehensive assessment of maternal, biochemical, biophysical, and ultrasound parameters during the first trimester. To the best of our knowledge, this is the first time that first-trimester characteristics of women with previous RPL have been assessed. However, several limitations must also be acknowledged. First, the heterogeneity of the RPL group, encompassing various etiologies and treatment regimens, complicates direct comparisons and necessitates cautious interpretation of results. Additionally, the relatively small sample size of the RPL group limits the generalizability of findings and may have limited the statistical power to detect subtle differences in first-trimester biomarkers or clinical outcomes. Finally, despite a higher rate of intrauterine demise in the RPL group, no other differences in maternal or neonatal outcomes were observed. This could be secondary to the higher proportion of parous women in the RPL group or to the widespread use of prophylactic treatment. Prospective studies aiming to clarify this are necessary.

In conclusion, our study contributes to the growing body of evidence on RPL by characterizing the first-trimester clinical profile of these women. Our findings support the view that women with previous RPL exhibit distinct maternal characteristics and are also at higher risk for intrauterine demise, despite finding no significant differences in first-trimester markers currently used in clinical practice. This underscores the need for intensified surveillance and tailored management strategies for this high-risk population until delivery, not just until first-trimester viability, even when first-trimester screening markers are within normal ranges. These may include increased early surveillance of fetal growth and placental function in the second and third trimesters, and consideration of early referral to maternal–fetal medicine specialists. Multidisciplinary care, involving maternal–fetal medicine specialists, hematologists, immunologists, and reproductive endocrinologists, is essential to optimize outcomes.

## Figures and Tables

**Table 1:** Maternal and pregnancy characteristics in women with and without recurrent pregnancy loss.

Maternal and Pregnancy Characteristics	No Recurrent Pregnancy Loss	Recurrent Pregnancy Loss	*p*
	n = 4298	n = 142	
Age (years)	33.9 (5.2)	37.8 (4.6)	<0.001
BMI (Kg/m^2^)	24.2 (4.7)	24.5 (4.6)	0.366
Smoking habit	344 (8)	9 (6.3)	0.635
Ethnic origin			
White	3091 (71.9)	109 (76.8)	
Latin-American	786 (18.3)	18 (12.7)	
Black	67 (1.6)	4 (2.8)	
Asian	107 (2.5)	2 (1.4)	0.396
South-Asian	150 (3.5)	5 (2.5)	
North-African	97 (2.3)	4 (2.8)	
Medical history			
Chronic hypertension	58 (1.3)	1 (0.7)	>0.999
Diabetes mellitus	43 (1)	1 (0.7)	>0.999
Renal disease	16 (0.4)	1 (0.7)	0.425
Autoimmune disease	18 (0.4)	2 (1.4)	0.133
Antiphospholipid syndrome	37 (0.9)	7 (4.9)	<0.001
Other thrombophilia	34 (0.8)	8 (5.6)	<0.001
Thyroid disorders	323 (7.5)	20 (14.1)	0.009
Assisted reproductive technologies	425 (9.9)	23 (16.2)	0.022
Egg donation	161 (3.7)	12 (8.5)	0.012
Obstetric history			
Nulliparous	2435 (56.7)	61 (43)	0.001
Parous with previous PE	106 (2.5)	6 (4.2)	0.173
Parous with previous SGA	63 (1.5)	5 (3.5)	0.065
Parous with previous GMD	74 (1.7)	6 (4.2)	0.042
Parous with previous PTB	83 (1.9)	4 (2.8)	0.360

Data are expressed as mean (SD) or n (%). BMI: Body mass index; PE: preeclampsia; SGA: small-for-gestational age; GDM: gestational diabetes mellitus; PTB: preterm birth.

**Table 2:** Comparison of pregnancy characteristics between women with and without recurrent pregnancy loss.

Pregnancy Characteristics	No Recurrent Pregnancy Loss	Recurrent Pregnancy Loss	*p*
	n = 4298	n = 142	
Biochemical markers			
GA at blood test	10.8 (1.1)	10.7 (1.0)	0.402
PAPP-A (MoM)	1.04 (0.73–1.50)	1.10 (0.73–1.53)	0.691
MAP (MoM)	1.03 (0.9–1.0)	1.03 (0.9–1.1)	0.809
First-trimester ultrasound			
GA at ultrasound	12.8 (0.6)	12.8 (0.5)	0.321
UtA-PI	1.64 (0.4)	1.66 (0.5)	0.516
UtA-PI (MoM)	1.04 (0.87–1.23)	1.06 (0.86–1.28)	0.696
Second-trimester ultrasound			
GA at ultrasound	20.8 (0.7)	20.7 (0.6)	0.184
UtA-PI	1.01 (0.3)	1.10 (0.4)	0.045
UtA-PI > 95th centile	170(4.2)	8 (6.3)	0.257
Screening for early-onset PE			
Very high-risk	220 (5.1)	15 (10.6)	0.011
Final high-risk	409 (9.5)	19 (13.4)	0.146
Prophylactic treatments			
Aspirin	410 (9.5)	34 (23.9)	<0.001
LMWH	3 (0.1)	21 (14.8)	<0.001

Data are expressed as mean (SD), n (%), or median (IQR). MoM: multiples of the median; GA: gestational age; PAPP-A: pregnancy-associated plasma protein-A; MAP: mean arterial pressure; UtA-PI: uterine artery pulsatility index; PE: preeclampsia; LMWH: low molecular weight heparin.

**Table 3:** Comparison of perinatal outcomes in women with and without recurrent pregnancy loss.

Pregnancy Outcome	No Recurrent Pregnancy Loss	Recurrent Pregnancy Loss	*p*
	n = 3777	n = 125	
Second-trimester miscarriage	53 (1.4)	8 (6.4)	0.001
Stillbirth	10 (0.3)	0 (0)	>0.999
Fetal sex			
Male	1950 (51.9)	69 (56.6)	0.357
Female	1806 (48.1)	53 (43.4)	
Birth weight (grams)	3248 (543)	3271 (551)	0.641
Overall PE	168 (4.5)	6 (4.8)	0.824
Early-onset PE	16 (0.4)	0 (0)	>0.999
Preterm PE	35 (0.9)	1 (0.8)	>0.999
Term PE	134 (3.5)	5 (4)	0.804
GA at birth	39.2 (3.2)	38.2 (6.0)	0.069
Cesarean delivery	925 (24.8)	33 (28)	0.449

Data are expressed as mean (SD) or n (%). PE: preeclampsia; GA: gestational age.

## Data Availability

The data presented in this study are available on request from the corresponding author. The data are not publicly available due to privacy or ethical restrictions.

## References

[1] Jauniaux E., Burton G.J. (2005). Pathophysiology of histological changes in early pregnancy loss. Placenta.

[2] Regan L., Rai R., Saravelos S., Li T.C. (2023). Gynaecologists the RC of O and. Recurrent MiscarriageGreen-top Guideline No. 17. BJOG Int. J. Obs. Gynaecol.

[3] Bender Atik R., Christiansen O.B., Elson J., Kolte A.M., Lewis S., Middeldorp S., Nelen W., Peramo B., Quenby S., ESHRE Guideline Group on RPL (2018). ESHRE guideline: Recurrent pregnancy loss. Hum. Reprod. Open.

[4] Dimitriadis E., Menkhorst E., Saito S., Kutteh W.H., Brosens J.J. (2020). Recurrent pregnancy loss. Nat. Rev. Dis. Primers.

[5] Habets D.H.J., Schiffer V.M.M.M., Kraneburg L.P.A., de Krom F.J.W., Gürtekin I., van Bree B.E., van Golde R.J.T., Wieten L., Spaanderman M.E.A., Al-Nasiry S. (2022). Preconceptional evaluation of women with recurrent pregnancy loss: The additional value of assessing vascular and metabolic status. BMC Pregnancy Childbirth.

[6] Turesheva A., Aimagambetova G., Ukybassova T., Marat A., Kanabekova P., Kaldygulova L., Amanzholkyzy A., Ryzhkova S., Nogay A., Khamidullina Z. (2023). Recurrent Pregnancy Loss Etiology, Risk Factors, Diagnosis, and Management. Fresh Look into a Full Box. J. Clin. Med..

[7] Gunnarsdottir J., Stephansson O., Cnattingius S., Akerud H., Wikström A.K. (2014). Risk of placental dysfunction disorders after prior miscarriages: A population-based study. Am. J. Obs. Gynecol..

[8] Bhattacharya S., McCall S.J., Woolner A.M.F. (2022). Recurrent pregnancy loss and subsequent preterm birth: Association or causation?. Fertil. Steril..

[9] Hautamäki H., Gissler M., Heikkinen-Eloranta J., Tiitinen A., Peuranpää P. (2025). Pregnancy and perinatal outcomes in women with recurrent pregnancy loss-A case-control study. Acta. Obs. Gynecol. Scand..

[10] Ausbeck E., Blanchard C., Neely C., Tita A., Szychowski J., Harper L. (2019). Perinatal outcomes in women with a history of recurrent pregnancy loss. Am. J. Obs. Gynecol..

[11] Serra B., Mendoza M., Scazzocchio E., Meler E., Nolla M., Sabrià E., Rodríguez I., Carreras E. (2020). A new model for screening for early-onset preeclampsia. Am. J. Obs. Gynecol..

[12] Gomez O., Figueras F., Fernandez S., Bennasar M., Martinez J.M., Puerto B., Gratacós E. (2008). Reference ranges for uterine artery mean pulsatility index at 11–41 weeks of gestation. Ultrasound Obs. Gynecol..

[13] Trilla C., Mora J., Ginjaume N., Nan M.N., Alejos O., Domínguez C., Vega C., Godínez Y., Cruz-Lemini M., Parra J. (2022). Reduction in Preterm Preeclampsia after Contingent First-Trimester Screening and Aspirin Prophylaxis in a Routine Care Setting. Diagnostics.

[14] Magnus M.C., Wilcox A.J., Morken N.H., Weinberg C.R., Håberg S.E. (2019). Role of maternal age and pregnancy history in risk of miscarriage: Prospective register based study. BMJ.

[15] Frick A.P. (2021). Advanced maternal age and adverse pregnancy outcomes. Best Pr. Res. Clin. Obs. Gynaecol..

[16] Mikwar M., MacFarlane A.J., Marchetti F. (2020). Mechanisms of oocyte aneuploidy associated with advanced maternal age. Mutat. Res. Rev. Mutat. Res..

[17] Santos T.D.S., Ieque A.L., de Carvalho H.C., Sell A.M., Lonardoni M.V.C., Demarchi I.G., de Lima Neto Q.A., Teixeira J.J.V. (2017). Antiphospholipid syndrome and recurrent miscarriage: A systematic review and meta-analysis. J. Reprod. Immunol..

[18] Alijotas-Reig J., Esteve-Valverde E., Anunciación-Llunell A., Marques-Soares J., Pardos-Gea J., Miró-Mur F. (2022). Pathogenesis, Diagnosis and Management of Obstetric Antiphospholipid Syndrome: A Comprehensive Review. J. Clin. Med..

[19] El Hachem H., Crepaux V., May-Panloup P., Descamps P., Legendre G., Bouet P.E. (2017). Recurrent pregnancy loss: Current perspectives. Int. J. Womens Health..

[20] de Jong P.G., Kaandorp S., Di Nisio M., Goddijn M., Middeldorp S. (2014). Aspirin and/or heparin for women with unexplained recurrent miscarriage with or without inherited thrombophilia. Cochrane Database Syst. Rev..

[21] Yan X., Wang D., Yan P., Li H. (2022). Low molecular weight heparin or LMWH plus aspirin in the treatment of unexplained recurrent miscarriage with negative antiphospholipid antibodies: A meta-analysis of randomized controlled trial. Eur. J. Obs. Gynecol. Reprod. Biol..

[22] Liu H., Shan Z., Li C., Mao J., Xie X., Wang W., Fan C., Wang H., Zhang H., Han C. (2014). Maternal subclinical hypothyroidism, thyroid autoimmunity, and the risk of miscarriage: A prospective cohort study. Thyroid.

[23] Wu Z., Cai Y., Xia Q., Liu T., Yang H., Wang F., Wang N., Yu Z., Yin C., Wang Q. (2019). Hashimoto’s thyroiditis impairs embryo implantation by compromising endometrial morphology and receptivity markers in euthyroid mice. Reprod. Biol. Endocrinol..

[24] Sovio U., Gaccioli F., Cook E., Charnock-Jones D.S., Smith G.C.S. (2024). Association between adverse pregnancy outcome and placental biomarkers in the first trimester: A prospective cohort study. BJOG.

[25] Luo Y., Zhou Y. (2023). Identification of novel biomarkers and immune infiltration features of recurrent pregnancy loss by machine learning. Sci. Rep..

[26] Rasmark Roepke E., Christiansen O.B., Källén K., Hansson S.R. (2021). Women with a History of Recurrent Pregnancy Loss Are a High-Risk Population for Adverse Obstetrical Outcome: A Retrospective Cohort Study. J. Clin. Med..

[27] Mohamedain A., Rayis D.A., AlHabardi N., Adam I. (2022). Association between previous spontaneous abortion and preeclampsia: A case-control study. BMC Pregnancy Childbirth.

[28] Clark K., Barton J.R., Istwan N., Rhea D., Desch C., Sibai A., Sibai B.M. (2012). PP179. The influence of prior abortion on rates of gestational hypertension/pre-eclampsia and spontaneous preterm delivery in nulliparous women. Pregnancy Hypertens.

